# Multi-institution analysis of racial disparity among African-American men eligible for prostate cancer active surveillance

**DOI:** 10.18632/oncotarget.25103

**Published:** 2018-04-20

**Authors:** Michael Dinizo, Weichung Shih, Young Suk Kwon, Daniel Eun, Adam Reese, Laura Giusto, Edouard J. Trabulsi, Bertram Yuh, Nora Ruel, Daniel Marchalik, Jonathan Hwang, Shilajit D. Kundu, Scott Eggener, Isaac Yi Kim

**Affiliations:** ^1^ Section of Urologic Oncology, Rutgers Cancer Institute of New Jersey and Rutgers Robert Wood Johnson Medical School, New Brunswick, NJ, USA; ^2^ Department of Biostatistics, Rutgers School of Public Health, New Brunswick, NJ, USA; ^3^ Department of Urology, Temple University, Philadelphia, PA, USA; ^4^ Department of Urology, Kimmel Cancer Center, Thomas Jefferson University, Philadelphia, PA, USA; ^5^ Division of Urology and Urologic Oncology, City of Hope National Medical Center, Duarte, CA, USA; ^6^ Department of Urology, Georgetown University, Washington, DC, USA; ^7^ Department of Urology, Feinberg School of Medicine, Northwestern University, Chicago, IL, USA; ^8^ Section of Urology, University of Chicago, Chicago, IL, USA

**Keywords:** prostate cancer, active surveillance, racial disparity

## Abstract

There is a significant controversy on whether race should be a factor in considering active surveillance for low-risk prostate cancer. To address this question, we analyzed a multi-institution database to assess racial disparity between African-American and White-American men with low risk prostate cancer who were eligible for active surveillance but underwent radical prostatectomy. A retrospective analysis of prospectively collected clinical, pathologic and oncologic outcomes of men with low-risk prostate cancer from seven tertiary care institutions that underwent radical prostatectomy from 2003–2014 were used to assess potential racial disparity. Of the 333 (14.8%) African-American and 1923 (85.2%) White-American men meeting active surveillance criteria, African-American men were found to be slightly younger (57.5 vs 58.5 years old; *p* = 0.01) and have higher BMI (29.3 v 27.9; *p* < 0.01), pre-op PSA (5.2 v 4.7; *p* < 0.01), and maximum percentage cancer on biopsy (15.1% v 13.6%; *p* < 0.01) compared to White-American men. Univariate and multivariate analysis demonstrated similar rates of upgrading, upstaging, positive surgical margin, and biochemical recurrence between races. These results suggest that single institution studies recommending more stringent AS enrollment criteria for AA men with a low-risk prostate cancer may not capture the complete oncologic landscape due to institutional variability in cancer outcomes. Since all seven institutions demonstrated no significant racial disparity, current active surveillance eligibility should not be modified based upon race until a prospective study has been completed.

## INTRODUCTION

Prostate cancer (PCa) affects more men than any other non-cutaneous malignancy. In 2017, 26,730 men are estimated to die from the disease [1]. Widespread prostate specific antigen (PSA) screening over the past two decades has coincided with an increase in the incidence of low-risk PCa that is unlikely to cause significant morbidity or mortality. Two separate PCa-screening studies have raised concerns on the harms associated with over-treating men with low-risk disease [2–4]. As a result, increasing emphasis has been placed on the role of active surveillance (AS) for men with low-risk PCa.

AS allows the opportunity to postpone or completely avoid definitive treatment in men with low-risk disease by periodically monitoring for evidence of disease progression [5]. Currently, there is no uniform consensus on the eligibility criteria for AS. The decision to enter an AS protocol is individually tailored to each patient's perceived cancer risk and personal preference, while also minimizing the chance of an undetected clinically significant tumor [6]. Regardless, the risk of understaging low-risk PCa is as high as 38% [7, 8]. It is therefore of critical importance to accurately identify those men who may benefit from immediate treatment versus those who may safely undergo conservative management and surveillance.

It is well established that there is a significant racial disparity in PCa. Multiple investigators have reported that African-American (AA) men are at increased risk for aggressive cancer [6, 9–13] and have a higher incidence of disease, worse pathologic outcomes, and higher mortality rates than White-American (WA) men [6, 14–17]. The underlying etiology of this racial disparity is likely heterogeneous and is thought to be due to a complex interaction of disease biology, access to healthcare, patient preferences, and socioeconomic factors. Due to such complexities, it remains unclear whether AS is an equally effective and valid management option for AA men.

Currently, there is a significant debate on whether the same AS eligibility criteria should be applied to all racial groups with low-risk PCa, or varied according to race [18]. Some studies have shown comparable rates of upgrading and upstaging, regardless of race, in men eligible for AS who underwent radical prostatectomy (RP) [19, 20]. On the contrary, an AS cohort found AA men to have a higher rate of disease progression compared to WA men [21]. In a separate study, we have found that AA men eligible for AS had worse clinicopathologic features on final surgical pathology compared to WA patients [22] and a recent large single institution study indicated that AA men had worse pathologic features following RP than a comparison group of WA men [23]. Accordingly, some investigators have suggested more stringent AS inclusion criteria specifically for AA men [24]. This study's aim was to clarify the feasibility of making the AS enrollment criteria for AA men with localized PCa more stringent, by retrospectively analyzing prospectively collected PCa databases from seven tertiary care institutions.

## RESULTS

A total of 2256 patients from seven institutions met the inclusion criteria (Table 1). Baseline clinical characteristics between the AA (*n* = 333) and WA (*n* = 1923) men are shown in Table 2. AA men had significantly higher BMI (29.3 v 27.9; *p* < 0.01) and were younger compared to the WA patients (57. v 58.5; *p* = 0.01). AA men also had higher pre-op PSA (5.2 v 4.7; *p* < 0.01). In addition, AA men were more likely to have multiple positive biopsy cores (53.1 v 47.6%; *p* < 0.01) and a higher rate of maximum percentage cancer on biopsy (15.1 v 13.6%; *p* < 0.01). However, WA men had a higher rate of T2a disease (11.7% v 4.2%, *p* < 0.01).

**Table 1:** Number of patients contributed by each participating institution

Institution	Number of Patients	
	WA	AA
**1**	267	75
**2**	40	37
**3**	68	47
**4**	85	47
**5**	669	74
**6**	663	13
**7**	131	40
**Total**	1923	333

**Table 2:** Patient characteristics

	White Men	AA Men	
Clinical Characteristics	(*n* = 1923)	(*n* = 333)	*p*-value
Age, years			**0.01^t^**
Mean	58.5	57.5	
95% CI	(58.2–58.8)	(56.7–58.2)	
Body Mass Index			**<0.01^t^**
Mean	27.9	29.3	
95% CI	(27.7–28.1)	(28.7–30.0)	
PSA			**<0.01^t^**
Mean	4.7	5.2	
95% CI	(4.6–4.8)	(5.0–5.4)	
Biopsy Gleason	6	6	
Clinical Stage (%)			**<0.01^t^**
T1a	11 (0.6)	1 (0.3)	
T1c	1686 (87.7)	318 (95.5)	
T2a	226 (11.7)	14 (4.2)	
Positive Cores (%)			0.1468^*^
1	1007 (52.4)	156 (46.9)	
2	558 (29.0)	112 (33.6)	
3	358 (18.6)	65 (19.5)	
Max. % Cancer per Core			**<0.05^t^**
Mean	13.6	15.1	
95% CI	(13.2–14.1)	(13.7–16.5)	
PSA Density			0.0176^t^
Mean	0.100	0.109	
95% CI	(.096–.104)	(.103–.114)	

^t^*T*-test.

^tt^Fisher's Exact.

^*^chisq test.

When pathologic characteristics were examined from the pooled data, there was no racial disparity in the rate of upstaging, upgrading, and positive surgical margin (*p* = 0.808, *p* = 0.1169, *p* = 0.1929 respectively) (Table 3). We next carried out a multi-variable analysis using variables that are readily available; race, age, BMI, PSA at diagnosis, single versus multiple positive biopsy cores and the maximum % cancer per core (Table 4). The results demonstrated that race was not associated with upgrading, upstaging, positive surgical margin rate, BCR when all institutions were combined.

**Table 3:** Pathologic outcome

Pathologic Characteristics	White Men	AA Men	*p*-value
	(*n* = 1923)	(*n* = 333)	
Upstage	7.3%	6.9%	0.808^*^
Pathologic Gleason			
≤6	1269 (66.0)	203 (61.0)	0.1982^*^
7	627 (32.6)	124 (37.2)	
≥8	27 (1.4)	6 (1.8)	
Upgrade (%)	34.0%	38.4%	0.1169^*^
Path Weight (g)	52.7 (51.8–53.6)	53.7 (51.31–56.2)	0.4276^t^
Positive Surgical Margin	12.1%	14.7%	0.1929^*^

^t^*T*-test.

^*^chisq test.

**Table 4:** Multi-variate analysis

Upgrade	Odds Ratio	Std. Error	*P*	95% CI: Lower	95% CI Upper
Race: AA vs WA	1.18	0.14	0.22	0.90	1.56
Age, years	1.03	0.01	<0.01	1.01	1.04
BMI	1.02	0.01	<0.03	1.0	1.05
PSA at diagnosis, ng/ml	1.14	0.03	<0.01	1.09	1.20
Biopsy Cores: Single vs Multiple Positive	1.50	0.10	<0.01	1.22	1.83
Max % Cancer Core	1.01	0.005	<0.01	1.00	1.02
**Upstage**	Odds Ratio	Std. Error	*P*	95% CI: Lower	95% CI Upper
Race: AA vs WA	0.82	0.27	0.44	0.48	1.38
Age, years	1.03	0.01	0.02	1.00	1.06
BMI	1.04	0.02	0.05	1.00	1.08
PSA at diagnosis, ng/ml	1.16	0.04	<0.01	1.06	1.26
Biopsy Cores: Single vs Multiple Positive	1.40	0.19	0.08	0.96	2.03
Max % Cancer Core	1.01	0.01	0.12	1.00	1.03
**Positive Surg. Margin**	Odds Ratio	Std. Error	*P*	95% CI: Lower	95% CI Upper
Race: AA vs WA	1.17	0.19	0.42	0.80	1.71
Age, years	1.00	0.01	0.92	0.98	1.02
BMI	1.04	0.02	<0.01	1.01	1.07
PSA at diagnosis, ng/ml	1.10	0.04	<0.01	1.02	1.19
Biopsy Cores: Single vs Multiple Positive	1.10	0.15	0.53	0.81	1.49
Max % Cancer Core	1.02	0.01	<0.01	1.00	1.03
**Biochemical Recurrence**	Hazard Ratio	Std. Error	*P*	95% CI: Lower	95% CI Upper
Race: AA vs WA	1.56	0.40	0.27	0.71	3.46
Age, years	1.04	0.02	0.09	0.99	1.09
BMI	0.96	0.04	0.28	0.89	1.03
PSA at diagnosis, ng/ml	1.14	0.08	0.10	0.98	1.32
Biopsy Cores: Single vs Multiple Positive	1.4	0.33	0.31	0.73	2.70
Max % Cancer Core	0.99	0.02	0.72	0.96	1.03

Because data for this study were collected from seven institutions, we next stratified the results by individual centers (Table 5). The results demonstrated large inter-institutional differences in rates of upstaging and upgrading in AA men, ranging from 0–10.8% and 27–47.3%, respectively. In addition, the positive surgical margin and BCR rates in AA men varied from 7.7% to 22.9% and 0% to 13.5%. Despite such wide range among the participating centers, no difference between AA and WA men was observed within each institution for the pathologic outcomes and BCR rate.

**Table 5:** Institutional characteristics

Institution	Upstage (%)			Upgrade%			Positive Surgical Margin			Biochemical Recurrence		
	Caucasian	African-American	*P*-value	Caucasian	African-American	*P*-value	Caucasian	African-American	*P*-value	Caucasian	African-American	*P*-value
**1**	6.70%	8.00%	0.7	29.20%	32.00%	0.64	11.20%	10.70%	0.89	3.47%	1.33%	0.3379
**2**	0.00%	10.80%	0.03	32.50%	27.00%	0.6	10.00%	21.60%	0.16	2.5%	0%	1
**3**	4.40%	8.50%	0.36	44.70%	39.70%	0.59	19.10%	14.90%	0.55	1.47%	0%	1
**4**	5.90%	10.60%	0.32	32.90%	36.20%	0.7	15.70%	19.20%	0.61	6.26%	13.51%	0.2818
**5**	7.00%	4.10%	0.33	40.50%	47.30%	0.26	12.40%	9.60%	0.48	2.19%	2.74%	0.6745
**6**	9.20%	7.70%	0.85	29.90%	46.20%	0.2	9.50%	7.70%	0.82	1.1%	0%	1
**7**	4.58%	0.00%	0.34	29.80%	37.50%	0.35	18.90%	22.90%	0.6	4.55%	10.34%	0.3624

## DISCUSSION

Our study of pooled data from seven tertiary institutions in the United States has one of the largest sample size to date on AA men eligible for AS (*n* = 333). AA men who were eligible for AS but chose surgery did not have significantly worse pathologic and oncologic outcome when compared to WA men. In addition, multi-variate analysis accounting for baseline clinical characteristics and institution did not demonstrate that race was an independent predictor of adverse outcomes. Collectively, these findings suggest that universally recommending modifications in AS criteria for AA men is premature at the present time.

The inter-institutional differences detected in the present study underscore the controversy surrounding AS and AA men. Previously, in a two-institution study, we have suggested that in men with low-risk PCa, AA men are more likely to have aggressive pathologic features than WA men [25]. Simultaneously, similar findings were reported using a more in-depth review of a single-institution database [23]. More recently, prospective AS cohorts with a relatively small sample size of AA men have supported the concept that low-risk PCa is inherently more aggressive in AA men [19]. Conversely, Jalloh *et al*. did not find a difference in the rates of upgrading and upstaging between AA and WA men following RP [19]. In this framework of contradicting results, our study provides the first clear evidence for significant inter-institutional differences in outcome for AA men with a low-risk prostate cancer.

In the present study, the rates of upstaging and upgrading in AA men among the seven participating institutions varied widely [0–10.8% (upstaging) and 27–47.3% (upgrading)]. In addition, the positive surgical margin and BCR rates in AA men varied from 7.7% to 22.9% and 0 to 13.5%, respectively. The reasons for these institutional variations in PCa outcomes are likely to be complex. Community and regional factors such as access to care, cancer screening, and lifestyle may significantly affect patient decisions and outcomes [26]. One compelling possibility is the subjective nature of pathologic interpretations. Indeed, inter-observer variability on RP specimens among pathologists has been reported to be as high as 30% [27]. An alternative explanation involves the heterogeneity among surgeons and subtle nuances of surgical techniques unique to each institution.

Results of the present study also shed light on the optimal design of potential clinical trials that will address racial disparity and AS. Herein, the pathologic and oncologic outcome in men with low-risk PCa varied widely. Therefore, any pooled data may reflect the bias of one institution if the accrual is disproportionate. Thus, optimal study design in the future should include a well-balanced accrual of patients from multiple sites. In short, any future prospective studies on AS in AA men should consist of multiple institutions with proportionate accrual.

Despite the large sample size reported to date on AS and racial disparity, this study has limitations. First, it is a retrospective study of patients who all underwent surgery. Thus, the study population in this investigation is not an AS cohort. Nevertheless, the findings should inform design of a prospective trial on AS in AA men. Second, due to the complexity of gathering data from multiple institutions, many potential confounding variables were unable to be accounted for; such as family history of cancer, PSA-velocity and socioeconomic status. These variables may have helped better account for institutional differences. Third, all institutions are tertiary care academic medical centers in major urban areas. Accordingly, the current findings may not be applicable to rural or community based practices due to selection bias. Fourth, the cohort is entirely from the pre MRI/US fusion biopsy era. Thus, a confirmatory study using the newer technology is currently being planned. Lastly, the lack of difference in BCR between the two races may be due to a significantly shorter follow-up period in AA men. We plan to analyze in detail the survival outcomes in our next study.

## MATERIALS AND METHODS

### Patients

The following seven institutions participated in the present study: Rutgers Cancer Institute of New Jersey (New Brunswick, NJ), City of Hope (Duarte, CA), Thomas Jefferson University (Philadelphia, PA), Temple University (Philadelphia, PA), Georgetown University (Washington, DC), Northwestern University (Chicago, IL), and University of Chicago (Chicago, IL). After obtaining the approval of the Institutional Review Board (IRB) from each site, prospectively collected data of men eligible for AS who underwent RP were analyzed retrospectively. All men who met Rutgers Cancer Institute's AS criteria for low-risk PCa were enrolled: (1) PSA ≤10 ng/ml, (2) 3 or fewer biopsy cores positive out of a minimum of 12-core biopsy, (3) biopsy Gleason score ≤6, (4) maximum biopsy core ≤50% cancer, and (5) clinical stage ≤T2a. All biopsy cores were taken using transrectal ultrasound guidance. Following RP, upgrading was defined as pathologic Gleason score ≥7 and upstaging as pathologic stage ≥ pT3. Biochemical recurrence (BCR) was defined as rise in PSA on two consecutive measurements with the last value ≥0.2 ng/ml. The number of patients contributed by each institution is shown in Table 1.

### Statistics

Clinical and pathologic characteristics were compared by race and between each institution. A multi-variable logistic regression model was used to evaluate the effect of race on pathologic variables while adjusting for readily available clinical data (year of surgery, race, age, BMI, clinical stage, PSA at diagnosis, single vs multiple positive biopsy cores and max % cancer per core) using Odds ratio. Differences in continuous variables were assessed using the *t* test. Categorical values were analyzed using the chi-square test and Fisher's exact test was used when frequencies were five or less. All statistical analyses were carried out using Stata SE 13 [28]. Only *p*-values < 0.05 were considered statistically significant.

## CONCLUSIONS

Further investigation, including prospective AS trials should be undertaken before any definitive judgment on AS and racial disparity can be made in men with PCa. In the meantime, race should not be a factor in counseling patients with low-risk PCa given that racial disparity in men eligible for AS was not observed in any of the seven participating sites in this study.

## Abbreviations

AA: African-American
WA: White-American
PCa: prostate cancer
AS: active surveillance
BCR: biochemical recurrence
